# Advancing Cr(VI) Electroreduction: A Redox Mediator to Catalyze the Electrochemical Reduction of Cr(VI) in Water While Preventing Fouling of Carbon Electrodes

**DOI:** 10.1021/acsorginorgau.3c00034

**Published:** 2023-11-06

**Authors:** Callie M. Stern, Malithi M. Abeythunga, Noémie Elgrishi

**Affiliations:** Department of Chemistry, Louisiana State University, Baton Rouge, Louisiana 70803, United States

**Keywords:** hexavalent chromium, PCET, water purification, electrocatalysis, electrode fouling

## Abstract

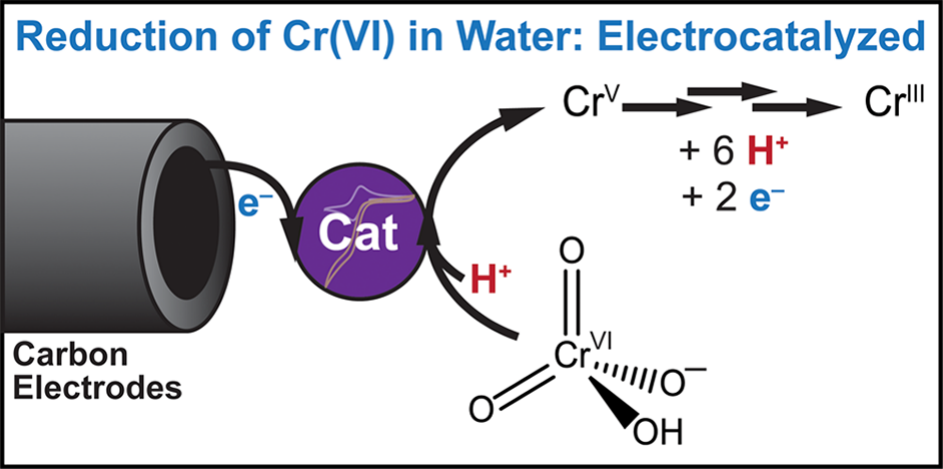

Hexavalent chromium is a contaminant of concern and is found in drinking water supplies. Electrochemical methods are well-suited to accomplish the reduction of toxic Cr(VI) to Cr(III). However, high overpotentials and plating of Cr(III) products on electrodes have stymied the development of efficacious purification methods. The Cr(VI) reduction reaction necessitates the transfer of multiple protons and electrons, which is accompanied by a high kinetic barrier. Following recent advances in the electrocatalytic energy storage community, we report that the use of [Fe(CN)_6_]^3–^ as a small molecular electrocatalyst not only diminishes the overpotential for Cr(VI) reduction on carbon electrodes by 0.575 V, but also prevents electrode fouling by mediating solution-phase homogeneous electron transfers.

## Introduction

Oxyanions are a ubiquitous class of compounds found in water, many of which present environmental challenges, depending on their concentrations. These include, for example, perchlorate, nitrate, nitrite, sulfate, phosphate, arsenate, and many more. Whether related to industry, defense, or agriculture, their leaching or improper handling has led to an increase in the prevalence of these deleterious oxyanions.^1,2^ This is apparent for example in the increased algae blooms leading to “dead zones” such as off the coast of the Mississippi delta in the Gulf of Mexico, caused in part by the agricultural runoff water rich in nitrite, nitrate, sulfate, and phosphate.^3^ Oxyanions present a challenge in drinking water, as well, with a famous example being chromate. Indeed, hexavalent chromium is a contaminant frequently found in water.^4−7^ Typically released from improper waste management, Cr(VI) is highly mobile, toxic, and difficult to remove from water.^7,8^ In contrast, Cr(III) in water is much more benign and can be precipitated depending on the pH. Electrochemical methods are ideal to affect the oxidation change, reducing toxic Cr(VI) to Cr(III) without using stoichiometric reagents or generating secondary waste. Electrochemical methods are being developed to both detect Cr(VI) in water and to reduce toxic Cr(VI) to more benign forms.^4,8−11^ Previous electrochemical methods had been confined to Cr(VI) reduction in highly acidic environments (pH < 3) and using mostly precious metal electrodes.^4,8,9,12^ We recently reported that inexpensive carbon electrodes can be effective for the detection and reduction of Cr(VI) in water using cyclic voltammetry.^13^ The reduction is initiated by a proton-coupled electron transfer (PCET) process in a pH range from 3 to 6, in agreement with data reported on Au electrodes in acidic media.^14^ The acid/base couple used to create the buffer in these experiments has an impact on the observed chemistry, even at a fixed pH,^15^ highlighting the noninnocence of buffers and electrolytes which continues to garner more attention in molecular electrocatalytic water purification and energy storage reactions.^16−20^ In particular, some buffers were shown to disfavor adsorption of Cr-containing species on the electrode surface on the time scale of cyclic voltammetry experiments.^15^ However, in bulk electrolysis conditions relevant to water purification, deposition is observed, which fouls the carbon electrodes and shuts down the electrochemical Cr(VI) reduction activity. Fe-based compounds as chemical reductants have long been used to treat solutions contaminated with Cr(VI) and as a method to decontaminate large bodies of water.^21^ Unfortunately, this method generates waste in the form of Fe–Cr sludges, which are difficult to handle and remove. Taking inspiration from this, sacrificial Fe electrodes have been used to promote similar transformations electrochemically, though still requiring stoichiometric amounts of Fe and generating Fe–Cr sludge waste.^22^ Herein we report on the use of Fe-based redox mediators to electrocatalytically reduce Cr(VI) and avoid the formation of secondary waste while preventing electrode fouling.

## Experimental Section

### General Considerations

All solutions were prepared with ultrapure Millipore deionized water obtained via a Milli-Q Advantage A10 Direct water purification system, with a resistivity of 18.2 MΩ cm at 25.0 °C. The following chemicals were used as received without further purification: potassium chromate (Alfa Aesar, 99.0%), potassium ferricyanide (Thermo Scientific Chemicals, ≥99.0%), potassium hexacyanoferrate(II) trihydrate (Strem Chemicals, Inc., ≥98.5%), citric acid disodium salt sesquihydrate (Alfa Aesar, 99.0%), sodium dihydrogen citrate (Alfa Aesar, 99.0%), hydrochloric acid (BDH Chemicals, 36.5–38%), and potassium hydroxide (BDH Chemicals, 85.0%). Potassium chloride (BDH Chemicals, 99.0–100.5%) was recrystallized from ethanol.

### General Buffer and Analyte Preparation

All solutions were freshly prepared. Citrate buffers were prepared by dissolving the required amounts of acid and base in water, followed by the adjustment with concentrated KOH or HCl to the appropriate pH as needed. A Mettler Toledo InLab MicroPro-ISM probe was used for pH measurements and calibrated before use with 10.01, 7.00, and 4.01 pH buffer solutions (Mettler Toledo) as received. Recrystallized KCl was added to all solutions as an electrolyte to control the ionic strength.

### Optical Methods

UV–vis absorbance spectra were collected by using a benchtop Ocean Optics DH-2000-BAL UV–vis-NIR light source coupled with optic fibers to an Ocean FX Spectrometer detector. Spectrosil quartz cuvettes (1 cm path length) were used for all measurements. Solutions, including the buffer and analyte, were prepared in a N_2_-filled glovebox (VAC MO-20) with degassed water. In each cuvette, 2.00 mL of buffer solution prepared in the glovebox was added, followed by aliquots from stock solutions of Cr(VI), Fe(II), and Fe(III) as required. Each cuvette was then sealed, and spectra were collected using the benchtop instrument.

### Electrochemical Methods

Following previously reported procedures,^13,15^ all electrochemical experiments were performed using a SP-300 Biologic potentiostat. Working electrodes used were 3 mm diameter glassy carbon disks (CH Instruments) or 100 PPI reticulated vitreous carbon (RVC, Duocel). All glassy carbon disks were freshly polished manually for 2 min with a slurry of 0.05 μm alumina powder (CH Instruments) in water on Microcloth polishing pads, then rinsed with water and sonicated for 20 s in water to remove any excess alumina powder. The electrodes were then briefly dried under N_2_. Multiple working electrodes were used: the capacitive currents of the electrodes were similar but not identical. For this reason, background capacitive currents for the specific electrodes are subtracted, and faradic currents are reported in analyses to compare data collected across the different electrodes. Recrystallized 1.00 M KCl was used as the supporting electrolyte for all experiments unless stated otherwise. Buffer solutions showed no electrochemical activity in the potential window scanned prior to the addition of analytes. Solutions were degassed with N_2_, after which the working electrodes were placed into the analyte solution for 30 s before the start of each scan.^13^ Disposable 20 mL borosilicate glass scintillation vials were used as electrochemical cells for cyclic voltammetry experiments. The vial was capped with a custom-made Teflon cap machined to have opening for the three electrodes and PTFE tubing for sparging.^23^ The typical volume of the solution was 5.00 mL. The working electrode was a 3 mm diameter glassy carbon electrode, the counter electrode was a 2 mm diameter platinum disk electrode (CH Instruments), and the reference electrode was Ag/AgCl 1.00 M KCl (CH Instruments) stored in 1.00 M KCl in water and rinsed before use.

Controlled-potential electrolysis (CPE) experiments were conducted in a two-compartment custom-made glass bulk electrolysis cell (pictures in Figure S6) adapted from prior work.^24,25^ All CPE experiments were performed under N_2_ with a constant stirring rate of 750 rpm used in the working electrode compartment to supply the electrode with fresh analyte. The working and counter electrode compartments, separated by a glass frit, have openings for electrodes and PTFE tubing. A coiled platinum wire (99.997%, 0.25 mm diameter) was used in the counter electrode compartment (5.00 mL). The working electrode compartment (10.0 mL) contained the working and reference electrodes. The working electrodes for CPE experiments were a fresh 30.0 × 10.0 × 6.00 mm^3^ 100 PPI RVC electrode cut to size, unless otherwise noted. The reference electrode was made following a published procedure.^26^ Briefly, a 1.00 mm diameter Ag wire (≥99.999% purity) was threaded through a rubber septum and cleaned by cycling between −0.30 and +1.20 V vs Ag/AgCl in 0.50 M H_2_SO_4_ at 0.100 V s^–1^. The clean Ag wire was then soaked in 0.10 M FeCl_3_, rinsed with water, and placed into a glass tube containing 1.00 M KCl in water sealed by a porous glass frit (Gamry Instruments) with heat shrinking PTFE tubing. The newly made Ag/AgCl reference electrode was stored in 1.00 M KCl and rinsed before use. The IUPAC convention is used to report currents and plot cyclic voltammograms (CVs).

## Results and Discussion

**Scheme 1:**
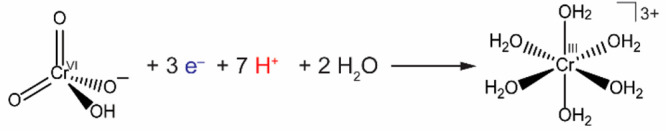
Expected Overall Reduction of Cr(VI) to Cr(III)

Based on the chromium Pourbaix diagram,^8,27^ in mildly acidic conditions and at low total chromium concentrations (10^–6^ M total dissolved chromium), the expected reduction of Cr(VI) is of the form in Scheme 1.

**Figure 1:**
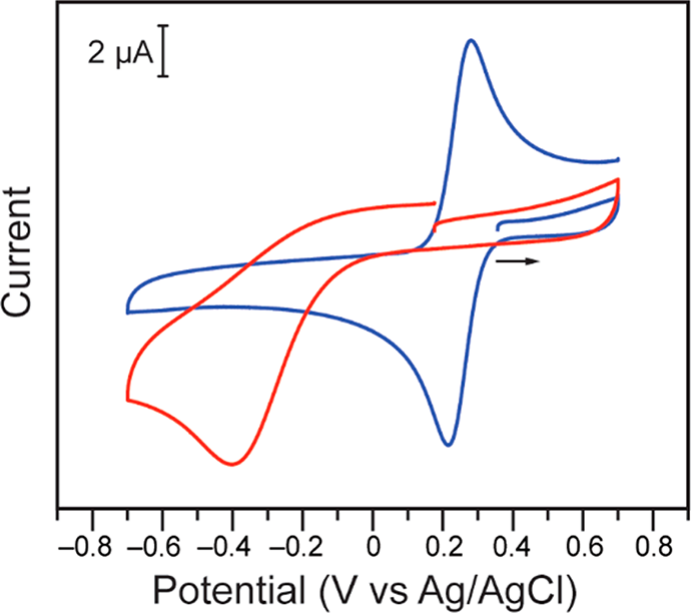
Cyclic voltammograms of 0.20 mM K_2_CrO_4_ (red) and 0.60 mM K_3_[Fe(CN)_6_] (blue) in 0.10 M citrate buffer at pH 4.00. Data were collected in 1.00 M KCl at scan rates of 0.100 V s^–1^ on 3 mm diameter glassy carbon electrodes. Plotting convention: IUPAC.

The associated standard reduction potential has been reported as approximately +1.35 V vs SHE,^4,8,28^ corresponding to +1.14 V vs Ag/AgCl.^26^ However, the reaction has a high kinetic barrier, as it necessitates the transfer of multiple protons and electrons. This leads to large overpotentials in practice: at pH 4 in water with a citrate buffer in the presence of KCl, the reduction peak potential of Cr(VI) is not observed until −0.40 V vs Ag/AgCl on carbon electrodes (Figure 1, red trace).

**Figure 2:**
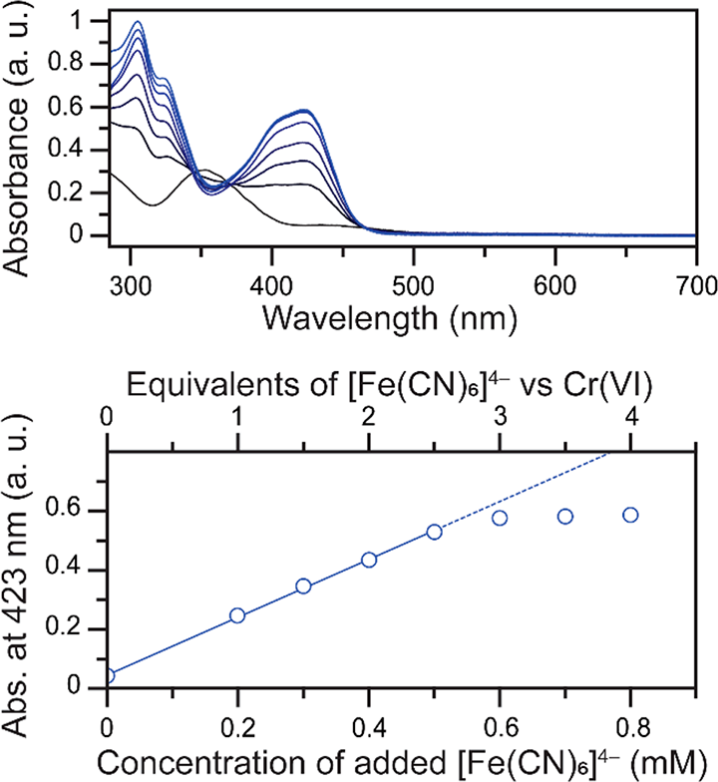
Top: Evolution (from black to blue) of the UV–vis spectra of 0.20 mM K_2_CrO_4_ after additions of 0, 0.20, 0.30, 0.40, 0.50, 0.60, 0.70, or 0.80 mM of K_4_[Fe(CN)_6_]. Data collected in a 0.10 M citrate buffer at pH 4.00 with 1.00 M KCl electrolyte. Bottom: evolution of the absorbance at 423 nm upon addition of K_4_[Fe(CN)_6_] with a linear fit for the first five data points (slope = 0.98; *r*^2^ = 0.998).

While the generated Cr(III) product is expected to be soluble based on the Pourbaix diagram,^8,27^ plating of insulating Cr(III) materials is frequently observed on electrodes, especially in bulk electrolysis conditions which shuts down activity.^9,14,29−34^ These Cr(III) products are reported as oxides or hydroxides, depending on specific conditions. In prior work, we have observed rapid fouling of carbon electrodes in bulk electrolysis conditions, with the presence of Cr(III) confirmed by X-ray photoelectron spectroscopy.^15^ Here we report the impact of using a redox mediator as a molecular electrocatalyst to promote the reduction of Cr(VI). The ferri/ferrocyanide couple was selected as a suitable redox mediator as this Fe-based organometallic complex is known to be water-soluble and to undergo a reversible 1-electron transfer in water. Specifically, the Fe(III)/Fe(II) couple, corresponding to [Fe(CN)_6_]^3–^/[Fe(CN)_6_]^4–^, in water at pH 4 with 1 M KCl electrolyte is observed at +0.25 V vs Ag/AgCl (Figure 1, blue trace). This is expected to be a sufficient driving force for a solution electron transfer from the reduced form [Fe(CN)_6_]^4–^ to Cr(VI) to occur. To test this hypothesis, the stoichiometric reaction between Cr(VI) and the reduced form [Fe(CN)_6_]^4–^ was first monitored by UV–vis spectroscopy (Figure 2). As [Fe(CN)_6_]^4–^ and Cr(VI) are mixed in solution, the intensity of the feature at 352 nm corresponding to Cr(VI) decreases (Figure 2, top).

Upon mixing, new absorption features appear at approximately 325 and 423 nm. These new features are attributed to [Fe(CN)_6_]^3–^ by comparison to a control sample of [Fe(CN)_6_]^3–^ in the same conditions (Figure S2). An isosbestic point is observed at 466 nm, which further supports the clean reduction of Cr(VI) by [Fe(CN)_6_]^4–^ and the generation of [Fe(CN)_6_]^3–^. We do not observe clean isosbestic points around 345 and 370 nm, in particular as excess [Fe(CN)_6_]^4–^ is added, because of the absorbance of both [Fe(CN)_6_]^4–^ and [Fe(CN)_6_]^3–^ in this region (Figure S2). Reduction of Cr(VI) would also generate Cr-containing products, which could also have absorbances in these regions.

The apparent stoichiometry of the reaction, based on the absorbance changes, supports the attribution of Cr(III) as the expected oxidation state of the reduction product. Indeed, the absorbance at 423 nm, where only the oxidized form [Fe(CN)_6_]^3–^ is expected to contribute to the intensity, shows a linear increase with addition of Fe(II), up to a saturation reached at a concentration of 0.60 mM K_4_[Fe(CN)_6_] (Figure 2, bottom). This supports a solution electron transfer from [Fe(CN)_6_]^4–^ to Cr(VI), reducing Cr(VI) and forming [Fe(CN)_6_]^3–^ in the process. Based on the saturation observed after 0.60 mM of [Fe(CN)_6_]^4–^ are added (3 equiv relative to K_2_CrO_4_), the overall stoichiometry corresponds to 3 [Fe(CN)_6_]^4–^ for every Cr(VI), following the overall transformation:
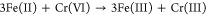
1

The stoichiometry observed also further supports the lack of redox reactions between Cr(VI) and the buffer, as the full 3 equiv of reduced iron centers is necessary for the reaction. Such reactions have been reported with some buffers, especially containing alcohols, in electrochemical conditions.^35^ Other reports have investigated the use of Cr(VI) as a chemical oxidant for alcohols in water, which is observed for primary and secondary alcohols, and critically requires much more acidic conditions for any reaction to take place.^36,37^ Here no redox reaction is observed between Cr(VI) and the buffer, which is consistent with prior work given the mildly acidic pH and the lack of primary or secondary alcohol functional group.^36,37^

**Figure 3:**
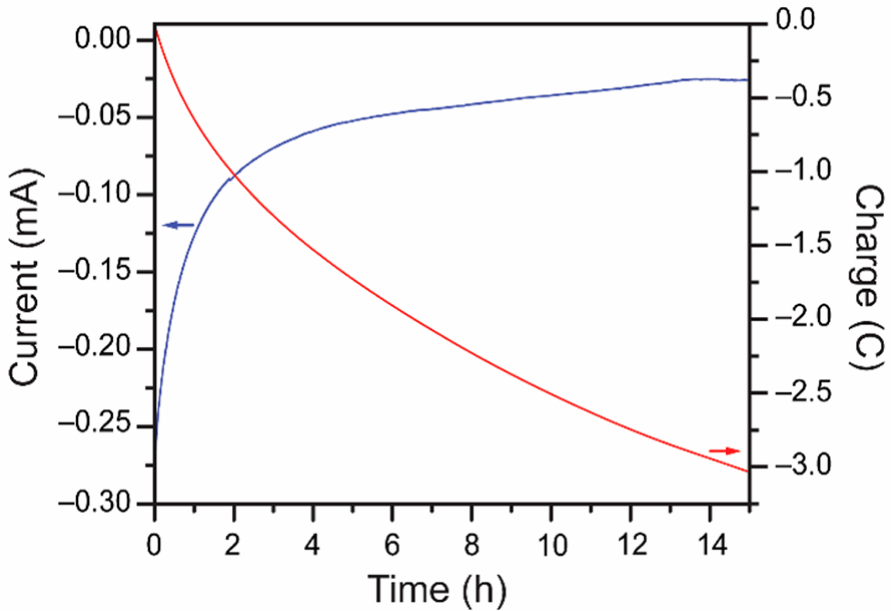
Evolution of the current (blue) and charge (red) during the CPE of 1.50 mM K_2_CrO_4_ in the presence of 50 μM K_3_[Fe(CN)_6_] at a fixed applied potential of +0.175 V vs Ag/AgCl.

The stoichiometric data support the theoretical possibility of the electrocatalytic reduction of Cr(VI) by the ferri/ferrocyanide couple in water. This was tested under controlled-potential electrolysis (CPE) conditions. Prior reports on glassy carbon electrodes at mildly acidic pH observed deposition on the electrode surface during Cr(VI) reduction in bulk electrolysis conditions, rapidly fouling the electrodes.^8,14,15^ Here, a CPE of a solution containing 1.50 mM Cr(VI) and 50 μM [Fe(CN)_6_]^3–^ was performed at a fixed applied potential of +0.175 V vs Ag/AgCl (Figure 3) and sustained currents were observed. Reticulated vitreous carbon (RVC) electrodes were used for these experiments.

The potential was chosen based on data obtained through cyclic voltammetry (Figure S1). During the CPE, minimal direct Cr(VI) reduction occurs at the potential chosen in the absence of [Fe(CN)_6_]^3–^ (Figures S3 and S4A), while the reduction of [Fe(CN)_6_]^3–^ to [Fe(CN)_6_]^4–^ is observed in the absence of Cr(VI) (Figure S4B). When Cr(VI) and [Fe(CN)_6_]^3–^ are mixed, the magnitude of the current observed increases significantly, and the currents are sustained over several hours, which supports Cr(VI) electrocatalytic reduction (Figure 3, blue).

The sustained currents observed throughout the 15 h of the CPE experiment underscore the different electrochemical system compared to prior reports: even after 15 h the current has still not reached the baseline (blue trace in Figure 3 compared to Figure S3). The rapid fouling of the carbon electrode observed for direct Cr(VI) reduction in the absence of a catalyst (Figure S5) is avoided in the presence of the electron mediator. While [Fe(CN)_6_]^3–^ reduction to [Fe(CN)_6_]^4–^ occurs at the electrode surface, Cr(VI) reduction can occur through a solution electron transfer from [Fe(CN)_6_]^4–^ to Cr(VI), away from the immediate vicinity of the electrode surface. The products formed do not plate on the electrode surface under these conditions, as evidenced by the sustained currents over several hours. While the current does decrease over time, it should be noted that Cr(VI) is consumed over time as the electrolysis progresses. This is apparent from the color changes throughout the electrolysis (Figure S6). Indeed, in Figure 3, the total charge passed was 3.036 C after 15 h. Using the equation *Q* = *nNF* allows us to calculate the number of electrons transferred to the solution. In this equation, *Q* is the charge passed in C, *n* is the number of electrons transferred per cycle, *N* is the number of moles of molecule reduced, and *F* is Faraday’s constant. For the 3-electron reduction of Cr(VI) to Cr(III), *n* = 3. This yields a maximum of *N* = 1.045 × 10^–5^ moles of Cr(VI) reduced based on the measured *Q* = 3.036 C after 15 h. At the start of the experiment, 1.50 × 10^–5^ moles of Cr(VI) were present in solution as determined based on the initial concentration of 1.5 mM of K_2_CrO_4_ and a working electrode compartment volume of 10.0 mL. Thus, based on the charge passed during the CPE, a maximum conversion of 70% was observed for Cr(VI) reduction to Cr(III) after 15 h. The initial amount of [Fe(CN)_6_]^3–^ in solution which is to be reduced to [Fe(CN)_6_]^4–^ during the process is negligible in comparison, accounting for only 0.048 C for a 1 electron reduction of the 50 μM K_3_[Fe(CN)_6_] present. These values demonstrate electrocatalytic turnover of [Fe(CN)_6_]^4–^ back to [Fe(CN)_6_]^3–^ during the bulk electrolysis, which enables the sustained reduction of Cr(VI). Based on the charge passed, over 62 electrocatalytic turnovers occurred during the CPE for every catalyst complex in the bulk solution.

Initial investigations of the mechanism were undertaken by using cyclic voltammetry. Typically, very large substrate equivalents would be used when studying the electrocatalytic reduction of small molecules.^38−40^ However, the rich and complex chemistry of Cr(VI) in water severely restricts the accessible substrate concentration range for these kinetic experiments.^8^ Indeed, an increase in total Cr(VI) in solution leads to the formation of dichromate following the equilibrium:
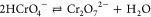
2

**Figure 4:**
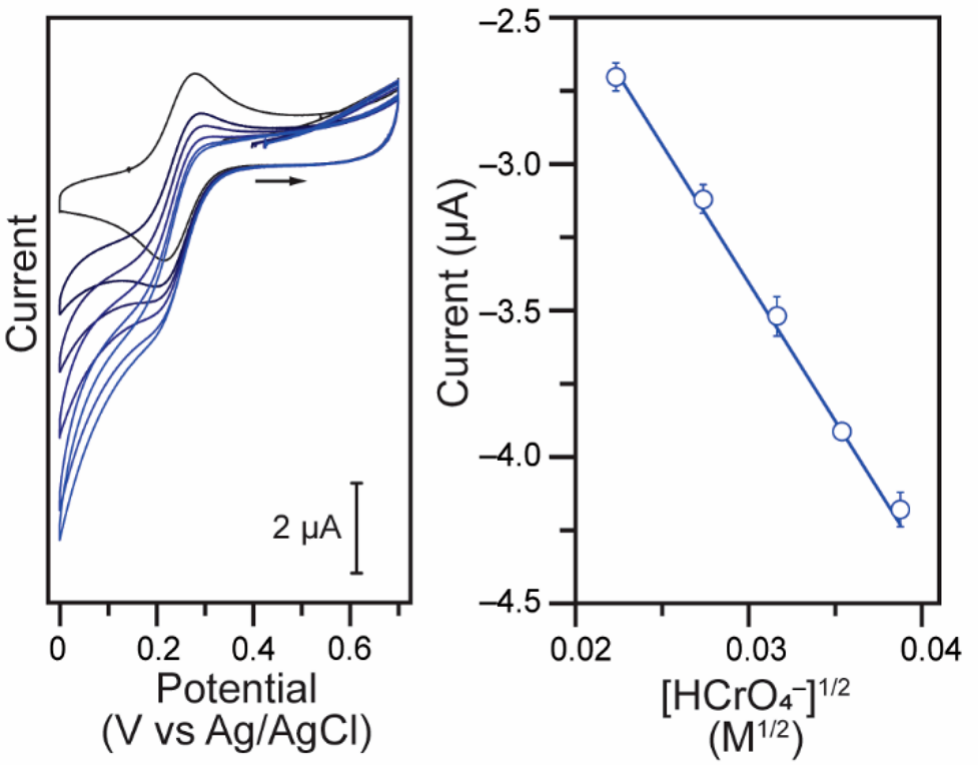
Left: representative cyclic voltammograms for 145 μM K_3_[Fe(CN)_6_] in the presence of (from black to blue) 0, 0.50, 0.75, 1.00, 1.25, and 1.50 mM K_2_CrO_4_ (10–30 equiv). Data were collected in a 0.10 M citric acid buffer at pH 4.00 in water with 1.00 M KCl electrolyte at scan rates of 0.100 V s^–1^. Plotting convention: IUPAC. Right: evolution of the catalytic faradaic current at +0.175 V vs Ag/AgCl as a function of the square root of the concentration of Cr(VI) with a linear fit (*r*^2^ = 0.998).

In cyclic voltammetry experiments, effects from the presence of dichromate were seen past 1.50 mM in similar conditions, though at pH 4.75.^13^ Thus, the total Cr(VI) concentration was kept at a maximum of 1.50 mM. This also ensures relevance to Cr(VI) reduction in contaminated water.^4,7,8,41^ Cyclic voltammetry data were collected for pH 4 solutions containing 145 μM [Fe(CN)_6_]^3–^ and increasing concentrations of Cr(VI). When both Cr(VI) and [Fe(CN)_6_]^3–^ are mixed, the ferricyanide reduction current increases, and the electrochemical feature loses reversibility. While the effects are small, they are noticeable and more pronounced as more Cr(VI) is added to the solution (Figure 4).

This supports the hypothesis that upon reduction of ferricyanide to ferrocyanide at the electrode, the in situ-generated [Fe(CN)_6_]^4–^ reduces Cr(VI). By doing so, it regenerates [Fe(CN)_6_]^3–^ and closes the catalytic cycle. As more equivalents of Cr(VI) are added, the voltammograms develop the characteristic catalytic s-shape (Figure 4). Higher concentrations of Cr(VI) also contribute to an increase in background currents near the switching potential due to the onset of direct Cr(VI) reduction at the electrode surface. The expected form of the plateau current for a catalytic s-shaped wave is^42,43^
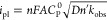
3where *F* is Faraday’s constant, *A* is the surface area of the electrode, *C*_P_^0^ is the concentration of the catalyst, *D* is the diffusion coefficient of the catalyst, and *n* represents the number of electrons transferred to the catalyst at the electrode, whereas *n*′ is the number of catalyst equivalents used per turnover.^42,43^

Given eq 3, the catalytic currents were plotted as a function of the square-root of the concentration of Cr(VI). The resulting plot shows a linear dependence (Figure 4, right), supporting a slow step that is first order in Cr(VI), with an expression of the observed rate constant of the form: *k*_obs_ = *k*[HCrO_4_^–^]. A similar experiment, varying the catalyst concentration while keeping the Cr(VI) concentration fixed, confirmed a first order in the catalyst (Figure S7).

Kinetic parameters can be estimated using the *i*_pl_/*i*_p_ equation, in which *i*_pl_ is the catalytic plateau current and *i*_p_ is the peak current for the catalyst in the absence of substrate:^42,43^
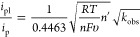
4

The exact form depends on the mechanism, with eq 4 having the underlying assumptions that the catalyst is reduced at the electrode, that other electron transfers occur in solution, and that the chemistry is limited by the first Cr(VI) reduction step. Should all electron transfers occur at the electrode instead, then *n*′ = *n* in eq 4. Plotting i_pl_/i_p_ as a function of  gives a linear plot (Figure S8). An estimate of the rate constant can be obtained from the slope, which here yields *k* = 4.9 × 10^2^ M^–1^ s^–1^ (Figure S8). We have previously shown that Cr(VI) reduction in similar conditions is initiated by the transfer of 1e^–^/1H^+^.^13,15^ The electron here is supplied by the catalyst, and the H^+^ is expected to be supplied by the buffer (pH 4 citrate). A solution chemistry report investigating the use of Cr(VI) as a chemical oxidant for Fe(II) at 303 K proposed a rate law which would translate to a rate constant of 8.5 × 10^2^ M^–1^ s^–1^ in our conditions (see calculation in the SI page SI-8).^44^ This estimated value is in good agreement with the CV data obtained.

**Scheme 2:**
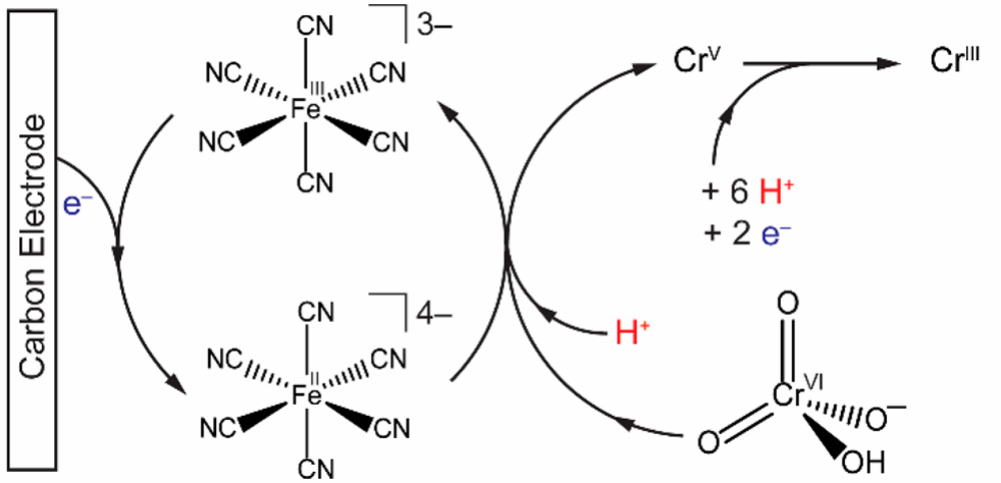
Proposed Catalytic Cycle

Overall, these data support the electrocatalytic reduction of Cr(VI) by [Fe(CN)_6_]^4–^ following a mechanism initiated by a PCET step. The PCET step could be either stepwise or concerted. The subsequent reduction of chromium containing species could involve fast solution electron transfers or disproportionation reactions.^8,14^ The proposed mechanism is summarized in Scheme 2.

This electrocatalytic process closes the loop and removes the need for stoichiometric reductants for Cr(VI) reduction in water.

## Conclusions

By using in situ electrogenerated [Fe(CN)_6_]^4–^ as a reductant for Cr(VI) instead of direct reduction of Cr(VI) at the electrode surface, electrode fouling is dramatically reduced, which improves the efficiency of the reduction of Cr(VI) to Cr(III). The introduction of solution electron transfers eliminates plating on the electrode surface. The overpotential required for Cr(VI) electroreduction is diminished by 0.575 V compared to a bare carbon electrode at pH 4 (Figure S1), controlled by the *E*_1/2_ of the [Fe(CN)_6_]^3–^/[Fe(CN)_6_]^4–^ couple. While the electrocatalytic activity is modest in terms of rates with the simple electron mediator chosen as a proof-of-concept, it is effective at reducing Cr(VI) to Cr(III), it diminishes the overpotential, and it avoids electrode fouling. The identification of a PCET process initiating Cr(VI) reduction allowed to use valuable insights gained from advances in mediating PCET processes in the context of small molecule activation and energy storage.^45−47^ Further methods to mediate the PCET processes required for efficient Cr(VI) reduction in water can be developed based on this work, and the kinetic parameters determined can be used as benchmarks for future homogeneous catalyst development. Current work focuses on developing electron and proton mediators to further reduce the overpotential and increase the reaction rate constant.

## Data Availability

The data underlying this study are available in the published article and its Supporting Information.

## Supporting Information Available

Supplementary data and analysis (cyclic voltammetry, CPE, UV–vis) (PDF)
