# Supplementary material for: Advancing Cr(VI) Electroreduction: A Redox Mediator to Catalyze the Electrochemical Reduction of Cr(VI) in Water While Preventing Fouling of Carbon Electrodes
Source: ACS Org Inorg Au. 2023 Nov 6;4(1):113–9. doi: 10.1021/acsorginorgau.3c00034 (PMC10853914; doi:10.1021/acsorginorgau.3c00034)
Supplement: Supplementary file 1 — gg3c00034_si_001.pdf [file gg3c00034_si_001.pdf]

Supporting Information

Advancing Cr(VI) Electroreduction: a Redox Mediator to Catalyze the Electrochemical Reduction of Cr(VI) in Water While Preventing Fouling of Carbon Electrodes

*Callie M. Stern, Malithi M. Abeythunga, and Noémie Elgrishi**

Department of Chemistry, Louisiana State University,

Baton Rouge, Louisiana 70803, United States

*Correspondence to: noemie@lsu.edu

Index *Page*

Cyclic Voltammetry Data

Control CVs of mixtures of Cr(VI) and $[Fe(CN)_6]^{3-}$ Figure S1 SI-3

Control UV-Vis Spectra

Control spectra of $[Fe(CN)_6]^{3-}$, $[Fe(CN)_6]^{4-}$, and Cr(VI) Figure S2 SI-3

Controlled-Potential Electrolysis (CPE) Data

CPE of the electrolyte Figure S3 SI-4

CPE of Cr(VI) and of $[Fe(CN)_6]^{3-}$ Figure S4 SI-4

CPE on 5 mm diameter glassy carbon electrodes Figure S5 SI-5

Setup and evolution of the CPE cell Figure S6 SI-5

Supporting Kinetic Data

Rate order in catalyst Figure S7 SI-6

Rate constant determination Figure S8 SI-7

Comparison of rate constant to stoichiometric reaction SI-8

References SI-8

Cyclic Voltammetry Data

Control CVs of mixtures of Cr(VI) and $[Fe(CN)_6]^{3-}$

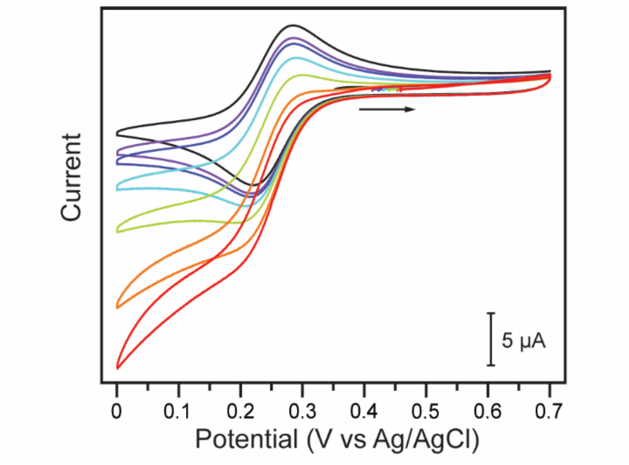

Figure S1: Cyclic voltammograms for 0.60 mM $K_3[Fe(CN)_6]$ in the presence of 0 (black, bottom), 0.20 (purple), 0.30 (blue), 0.60 (cyan), 1.20 (green), 2.40 (orange), and 3.60 (red, top) mM of K_2CrO_4 (0.3 to 6 equivalents). Data collected in a 0.10 M citric acid buffer at pH 4.00 in water with 1.00 M KCl electrolyte at scan rates of 0.100 V s^{-1}. Plotting convention: IUPAC.

Control UV-Vis Spectra

Control spectra of $[Fe(CN)_6]^{3-}$, $[Fe(CN)_6]^{4-}$, and Cr(VI)

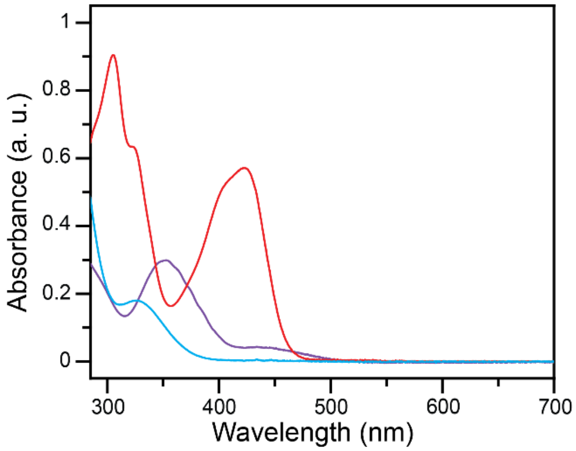

Figure S2: UV-Vis spectra of 0.20 mM K_2CrO_4 (purple), 0.60 mM $K_3[Fe(CN)_6]$ (red), and 0.60 mM $K_4[Fe(CN)_6]$ (blue) in a 0.10 M citate buffer at pH 4.00 in 1.00 M KCl. Data collected in individual cuvettes under N_2 atmosphere.

Controlled-Potential Electrolysis (CPE) Data

CPE of the electrolyte

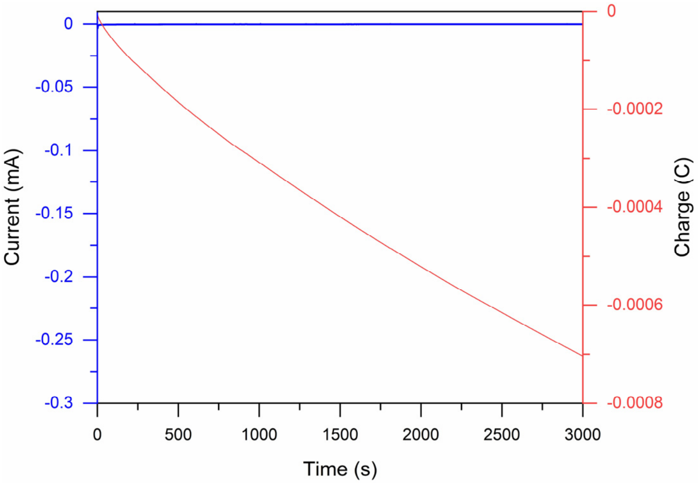

Figure S3: Evolution of current (blue) and charge (red) during the bulk electrolysis of the electrolyte at a constant potential of +0.175 V vs Ag/AgCl. Data collected on a reticulated vitreous carbon electrode in a 0.10 M citrate buffer at pH 4.00 with 1.00 M KCl electrolyte.

CPE of Cr(VI) and of $[Fe(CN)_6]^{3-}$

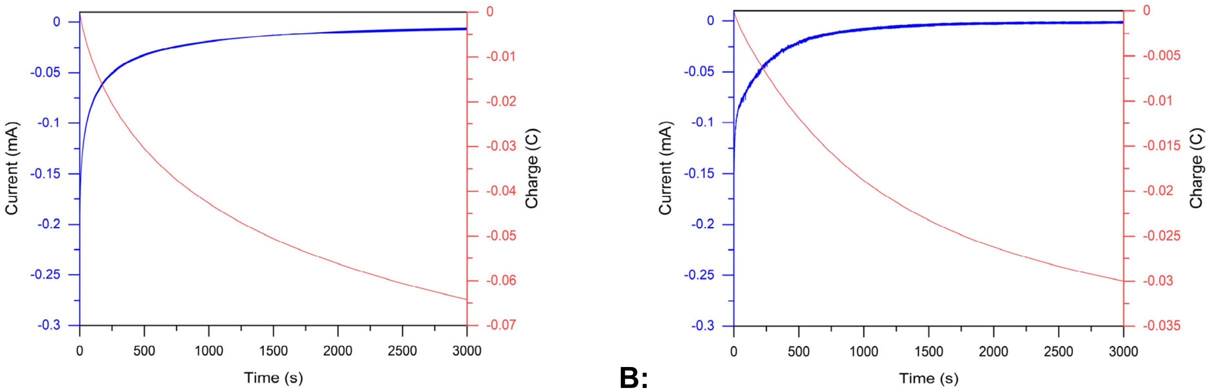

A: **B:**

Figure S4: **A:** Evolution of current (blue) and charge (red) during the bulk electrolysis of 1.50 mM K_2CrO_4 at a constant potential of +0.175 V vs Ag/AgCl. Data collected on a reticulated vitreous carbon electrode in a 0.10 M citrate buffer at pH 4.00 with 1.00 M KCl electrolyte. **B:** Evolution of current (blue) and charge (red) during the bulk electrolysis of 50 μM $K_3[Fe(CN)_6]$ at a constant potential of +0.175 V vs Ag/AgCl. Data collected on a reticulated vitreous carbon electrode in a 0.10 M citrate buffer at pH 4.00 with 1.00 M KCl electrolyte.

CPE on a 5mm glassy carbon electrode of the electrolyte, of Cr(VI), and of $[Fe(CN)_6]^{3-}$

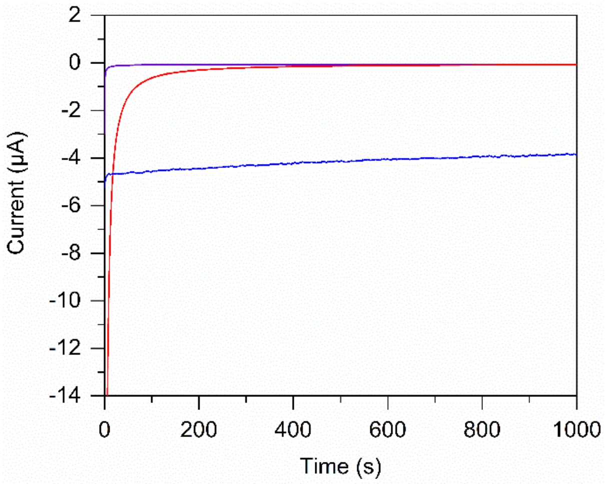

Figure S5: Evolution of current over time during controlled potential electrolysis experiments on a 5 mm diameter glassy carbon electrode polished and pretreated as described for CV data. Three experiments: CPE in a 0.10 M citrate buffer at pH 4.00 with 1.00 M KCl electrolyte at a constant applied potential of –0.44V vs Ag/AgCl in the absence (purple trace) or presence (red trace) of 1.50 mM K_2CrO_4. These show rapid fouling of the electrode after only a fraction of the Cr(VI) is reduced. Comparison to CPE of an identical solution with now added 50 μM $K_3[Fe(CN)_6]$ at a constant potential of +0.175 V vs Ag/AgCl showing sustained reduction currents with no evidence of electrode fouling.

Setup and evolution of the CPE cell

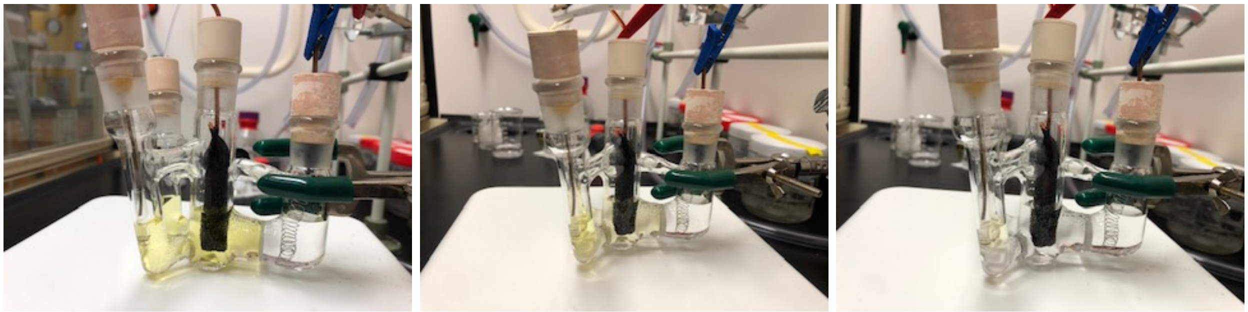

Figure S6: Picture evolution at start (left), 7 hours (middle), and 15 hours (right) for the bulk electrolysis of 1.50 mM K_2CrO_4 in the presence of 50 μM $K_3[Fe(CN)_6]$ at a constant potential of +0.175 V vs Ag/AgCl. Data collected on a reticulated vitreous carbon electrode in a 0.10 M citrate buffer at pH 4.00 with 1.00 M KCl electrolyte.

Supporting Kinetic Data

Rate order in catalyst

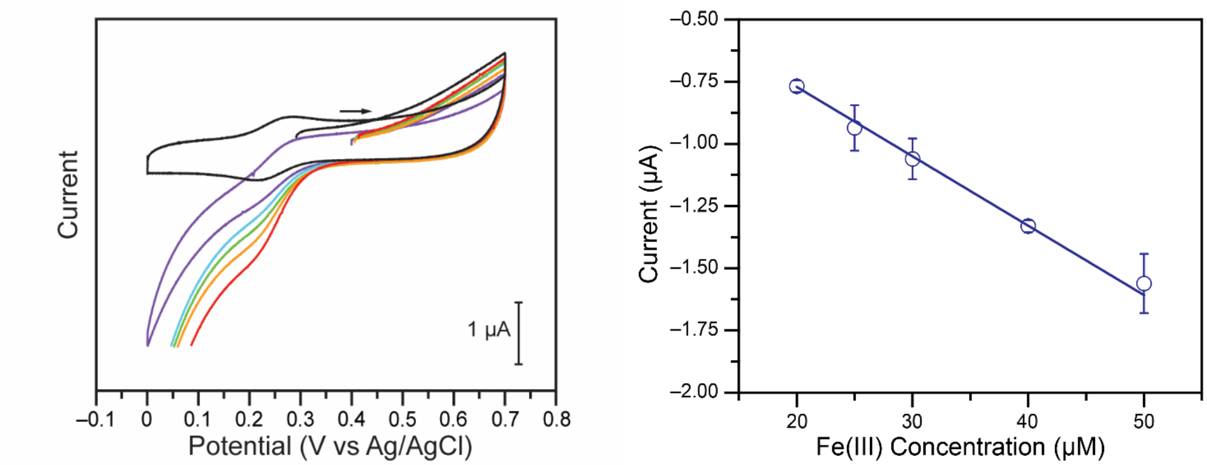

Figure S7: Cyclic voltammogram (left) of 20 µM $K_3[Fe(CN)_6]$ in the absence (black) and presence (purple) of 1.50 mM K_2CrO_4. Subsequently, additions of Fe(III) increased the concentration of $K_3[Fe(CN)_6]$ to 25 µM (blue), 30 µM (green), 40 µM (orange) and 50 µM (red). Data collected in a 0.10 M citate buffer at pH 4.00 with 1.00 M KCl electrolyte at scan rates of 0.100 V s^{-1} on 3 mm diameter glassy carbon electrodes. Right: linear fit of the evolution of faradaic currents at +0.200 V vs Ag/AgCl with standard deviations as a function of the concentration of $K_3[Fe(CN)_6]$ added (r^2 = 0.999). Plotting convention: IUPAC.

Rate constant determination

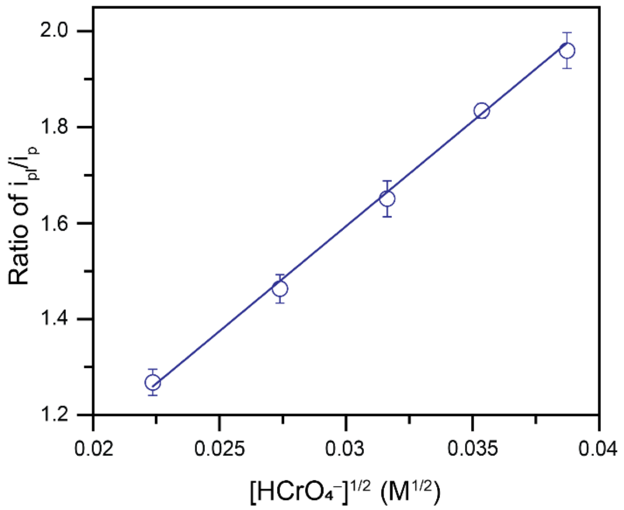

Figure S8: Ratio of the faradaic catalytic plateau current i_{pl} (measured at +0.175 V vs Ag/AgCl) at various concentrations of Cr(VI) divided by the peak current for the Fe(III)/Fe(II) couple in the absence of Cr(VI) i_p. Data collected from the CV traces in Figure 4 of the main text. The slope of the linear fit is 43.7 ± 1.1 (r^2 = 0.999) which translates to *k* values of 4.9 10^2 M^{-1} s^{-1} following the calculations detailed below:

$$\frac{i_{pl}}{i_p} = \frac{1}{0.4463}\sqrt{\frac{RT}{nF\upsilon}n'\sqrt{k_{obs}}}$$

$$\frac{i_{pl}}{i_p} = \frac{1}{0.4463}\sqrt{\frac{RT}{nF\upsilon}n'\sqrt{k}} \times \sqrt{[HCrO_4^-]}$$

Calling the slope of the graph A yields: $A = \frac{1}{0.4463}\sqrt{\frac{RT}{nF\upsilon}n'\sqrt{k}}$

$$k = (A \times 0.4463)^2 \frac{nF\upsilon}{RTn'}$$

A value of *k* of 4.8 10^2 M^{-1} s^{-1} is obtained using the following parameters:

R = 8.314 J mol^{-1} K^{-1}

T = 298 K

F = 96485 C mol^{-1}

υ = 0.1 V s^{-1}

n = 1

n' = 3

Comparison of electrocatalytic rate constant to stoichiometric reaction

Howlett and Sulfab proposed that the stoichiometric oxidation of Cr(VI) by Fe(II) follows:[1]

We have previously shown that Cr(VI) reduction in similar conditions is gated by the slow transfer of $1e^-/1H^+$.[2] The electron here is supplied by the catalyst, and the H^+ by the buffer (pH 4 citrate). A solution chemistry report investigating the use of Cr(VI) as a chemical oxidant for Fe(II) proposed an overall rate law of:[1]

$$-\frac{d[HCrO_4^-]}{dt} = [Fe(CN)_6^{4-}][HCrO_4^-][H^+](k_0 + k'[K^+])$$

Where k_0 = 2.0 10^6 M^{-2} s^{-1} and k' = 6.5 10^6 M^{-3} s^{-1} at a temperature of 303 K.

In our conditions, the concentration of potassium ions is constant and very large at 1.00 M to minimize ionic strength variations. This would translate to a rate constant $k'' = k_0 + k'[K^+]$ of:

k'' = 2.0 10^6 M^{-2} s^{-1} + 6.5 10^6 M^{-3} s^{-1} × 1 M

k'' = 8.5 10^6 M^{-2} s^{-1}

The cyclic voltammetry experiments were conducted at pH 4, in the presence of a buffer. $[H^+]$ is expected to be constant, at $[H^+] = 10^{-\mathrm{pH}} = 10^{-4}\ M$.

This would yield a rate constant of $k = [H^+] \times k''$:

$k =$ 8.5 10^2 M^{-1} s^{-1}

This estimated value is the same order of magnitude as the data obtained from cyclic voltammetry.

References

(1) Howlett, K. E.; Sulfab, Y. Kinetics and Mechanisms of the Reaction between Chromium(VI) and Hexacyanoferrate(II) in Aqueous Solution. *Inorganica Chim. Acta* **1976**, *17*, 129–133. https://doi.org/10.1016/S0020-1693(00)81970-2.

(2) Stern, C. M.; Hayes, D. W.; Kgoadi, L. O.; Elgrishi, N. Emerging Investigator Series: Carbon Electrodes Are Effective for the Detection and Reduction of Hexavalent Chromium in Water. *Environ. Sci. Water Res. Technol.* **2020**, *6* (5), 1256–1261. https://doi.org/10.1039/D0EW00146E.
